# Supplementary material for: Analysis and optimization of 15-minute community life circle based on supply and demand matching: A case study of Shanghai
Source: PLoS One. 2021 Aug 31;16(8):e0256904. doi: 10.1371/journal.pone.0256904 (PMC8407582; doi:10.1371/journal.pone.0256904)
Supplement: Attachment — Submitted filename: response.docx [file pone.0256904.s001.docx]

Dear Prof. Chen and Reviewers:

Thank you for your letter and for the reviewers’ comments concerning our manuscript entitled “**Analysis and optimization of 15-minute community life circle based on supply and demand matching: A case study of Shanghai**” **(ID: PONE-D-21-14500)**. Those comments are all valuable and very helpful for revising and improving our paper, as well as the important guiding significance to our researches. We have studied comments carefully and have made correction which we hope meet with approval. Revised portion are marked in red in the paper. The main corrections in the paper and the responds to the reviewer’s comments are as following:

**Responds to journal requirements:**

As required by the journal, all pictures and content should be published in compliance with CC4.0. Therefore, we replaced the boundary data of the maps included in the article (Figures 1.2.4.5 and 6) with open-access Nature Earth vector boundary data and added a text description of the source of the image in the corresponding title of the image.

**Responds to the reviewer’s comments:**

**Reviewer #1:**

**1.** **Response to comment:**

The research background is kind of insufficient. The readers may need to know more about the concept of the “15-minute community life cycle”. Maybe adding a paragraph describing current policies and similar policies in other cities/countries may help the readers better understand the concept. I would also recommend the authors to add more content in key factors of the concept (for example, what kinds of facilities are important for this concept? It might be better to explain the design of the models), so the readers will have no problems in understanding the following analysis.

**Response:**

After considering your suggestions, we re-written the **introduction**, including its content and the order of paragraphs. **In the first paragraph,** we have supplemented the description of the concept of Japanese community life circle, such as **‘**that is, life centers …… according to a certain proportion,**’;** We have also expanded the description of the development of theoretical research on community life circles in European and American countries **in the second paragraph,** such as ‘In European and American countries, …… with more complete life services[5].**’,** and other existing policies related to the 15-minute community living circle, such as ‘In the post-epidemic era, …… as a pandemic economic recovery tool[7].’ In addition, as you suggested, we have also added a description of the factors that should be emphasized in the concept of 15-minute community living circle, such as ‘Based on the existing domestic and foreign theoretical and empirical research on the 15-minute community living circle, ……, but also the characteristics of the population and the population's demand for basic service facilities.’ **in the penultimate paragraph of this section.**

**2. Response to comment:** It may be clearer to add a data section in materials and methods to introduce the data sources systematically.

**Response:**

After considering your suggestions, we think it is very appropriate to write the description of the data in the method and material section of the article as a separate subsection. Therefore, **in the materials and methods section, we merged the original three parts of "POI", "urban road network" and "population" into the "data" subsection.** We also **added detailed descriptions to the existing data descriptions in the text.**

**3. Response to comment:**

This research has the potential to be further developed, while the experience in Shanghai may be suitable for other cities in China or even in other countries. Maybe the author could add more general discussions on policy implications that are not limited to Shanghai's local context.

**Response:**

After considering your suggestions, we have supplemented the relevant content **in the optimization policy section**, and have also made corresponding changes to the relevant titles of the chapters. **In the ‘15min-CLC optimization strategy based on the perspective of supply improvement’ part,** we added ‘The implementation of the community business model has also been initially explored at home and abroad. ……, quantity and use efficiency of shopping service facilities.’ **to** illustrate the domestic and foreign discussions on the development of community business models and the relevant recommendations of this article on the promotion of this model; **In the next part,** we supplemented the existing case of the 15-minute community life circle open sharing plan, and expounded on the implementation of the suggestions proposed in this article for the optimization of the existing plan, such as ‘The functional sharing and opening of community space have always been the direction of the 15min-CLC. ……, to plan a 15-minute community living circle in a way of shared use and joint planning.’.

**Reviewer #2:**

**1. Response to comment:**

This paper needs to engage with the wider readership of the journal. How to link the findings/conclusions in this paper with the previous findings/conclusions from other countries?

**Response:**

As you suggested, we have added some existing policies and cases of other countries regarding the 15-minute community living circle in the optimization policy section, and supplemented the future optimization directions for these policies and cases proposed in this article, such as **‘**In the future, the hierarchical spatial layout of physical shopping service facilities can be optimized ……, quantity and use efficiency of shopping service facilities.’ in the part of **‘15min-CLC optimization strategy based on the perspective of supply improvement’** and **‘**In the future, for various regions at home and abroad, ……, to plan a 15-minute community living circle in a way of shared use and joint planning.’ in the part of ‘**15min-CLC optimization strategy based on the perspective of demand matching**’.

**2. Response to comment:**

In the method section. It is unnecessary to explain the meanings of $W_{k}$ and $D_{k}$ twice.

**Response:**

We are very sorry for the negligence of the repeated explanation in the text. For this **we have kept the explanation of the parameters** $\boldsymbol{W}_{\boldsymbol{k}}$ **and** $\boldsymbol{D}_{\boldsymbol{k}}$ **that appeared at the beginning (in the ‘Weighted kernel density estimation and 15min-CLC infrastructure service facilities mixed diversity index’),** and deleted the explanation of $W_{k}$ and $D_{k}$ that appeared in the "Population matching measurement model and 15min-CLC infrastructure service facility population supporting index".

**3. Response to comment:**

The color distribution of Figure 5 is unclear. Please change the color to make it easy to identify areas with high, medium, and low values.

**Response:**

It is really true as you suggested that the color distribution in Figure 5 should be optimized. Therefore, **we changed the color bar, adjusted the color configuration, and remade Figure 5.**

Finally, all the above changes and other changes to the format and writing have been marked in red in the text. We appreciate for Professor Chen and reviewers’ warm work earnestly, and hope that the correction will meet with approval.

Yours sincerely,

Liangxu Wang

Corresponding author:

Name: Liangxu Wang

E-mail: wangliangxu@shnu.edu.cn
